# Effects of Perioperative Dexmedetomidine on Immunomodulation in Uterine Cancer Surgery: A Randomized, Controlled Trial

**DOI:** 10.3389/fonc.2021.749003

**Published:** 2021-11-16

**Authors:** Jin Sun Cho, Kieun Seon, Min-Yu Kim, Sang Wun Kim, Young Chul Yoo

**Affiliations:** ^1^ Department of Anesthesiology and Pain Medicine, Yonsei University College of Medicine, Seoul, South Korea; ^2^ Anesthesia and Pain Research Institute, Yonsei University College of Medicine, Seoul, South Korea; ^3^ Department of Obstetrics and Gynecology, Division of Gynecologic Oncology, Institute of Women’s Life Science, Yonsei University College of Medicine, Seoul, South Korea

**Keywords:** dexmedetomidine, immunity, interferon-γ, natural killer cell, uterine cancer

## Abstract

**Objective:**

Dexmedetomidine has sympatholytic, anti-inflammatory, and analgesic effects and may exert anti-tumor effect by acting on α2A adrenoreceptor. We investigated whether perioperative dexmedetomidine preserves immune function in patients undergoing uterine cancer surgery.

**Methods:**

One hundred patients were randomly assigned to the control or dexmedetomidine groups (50 patients each). Dexmedetomidine was infused at rates of 0.4 μg/kg/h intraoperatively and 0.15 μg/kg/h during the first 24 h postoperatively. The primary outcome was natural killer (NK) cell activity, which was measured preoperatively and 1, 3, and 5 days postoperatively. The inflammatory response was measured by interleukin-6, interferon-γ, and neutrophil/lymphocyte ratio, and pain scores and opioid consumption were assessed. Cancer recurrence or metastasis and death were evaluated 2 years postoperatively.

**Results:**

NK cell activity decreased postoperatively in both groups and changes over time were not different between groups (*P*=0.496). Interferon-γ increased postoperatively in the dexmedetomidine group, whereas it maintained at the baseline value in the control group. Change in interferon-γ differed significantly between groups (*P*=0.003). Changes in interleukin-6 and neutrophil-lymphocyte ratio were comparable between groups. Both pain score with activity during the first 1 h and opioid consumption during the first 1–24 h postoperatively were lower in the dexmedetomidine group. Rates of cancer recurrence/metastasis (16.3% vs. 8.7%, *P*=0.227) and death within 2 years postoperatively (6.7% vs. 2.2%, *P*=0.318) were not different between groups.

**Conclusions:**

Perioperative dexmedetomidine had no favorable impacts on NK cell activity, inflammatory responses, or prognosis, whereas it increased interferon-γ and reduced early postoperative pain severity and opioid consumption in uterine cancer surgery patients.

## Introduction

Although surgical resection is the main and curative treatment for solid tumors, the spread of tumor cells in the blood and lymphatic system might occur by surgical manipulation (1). Surgical trauma-induced systemic stress and inflammatory responses and the use of anesthetics and opioid analgesics impair immune function (2). This perioperative immunosuppression may predispose already immunocompromised cancer patients further vulnerable to tumor growth and spread. Whether residual tumor cells adversely affect patient’s outcome depends on the balance between the host’s immune defenses against tumor and factors promoting tumor cell survival and growth.

Dexmedetomidine is a highly selective α2 adrenoreceptor agonist and has broad pharmacologic effects including anesthesia, analgesia, sedation, and anxiolysis (3). Perioperative dexmedetomidine attenuates stress responses and reduces pain and opioid requirement in the perioperative periods (4–6). In addition, dexmedetomidine has sympatholytic and anti-inflammatory effects (5, 7). Perioperative immunosuppression is characterized by suppressed cell-mediated immunity and excessive pro-inflammatory responses (8). Dexmedetomidine has been demonstrated to preserve natural killer (NK) cell function, which is a critical part of innate immunity, and reduce pro-inflammatory cytokines in both experimental and clinical settings (4, 7). Despite possible beneficial effects of dexmedetomidine on immunity, its immunomodulatory role in cancer surgery has not been established.

Gynecologic cancer contributes significantly to the morbidity and mortality of females worldwide (9), and cervical and endometrial cancers are the most frequent gynecologic malignancies (10). In this randomized, controlled trial, we investigated the effect of dexmedetomidine on immunomodulation in women undergoing uterine cancer surgery. Based on the immunomodulatory effects of dexmedetomidine, we hypothesized that dexmedetomidine would attenuate the immunosuppression during the critical perioperative period.

## Materials and Methods

This study was approved by the Institutional Review Board and Hospital Research Ethics Committee of Severance Hospital, Yonsei University Health System, Seoul, Korea (#4-2015-0453) and registered at ClinicalTrials.gov (NCT02896413). Inclusion criteria were women 20–65 years old, who had American Society of Anesthesiologists (ASA) physical status classification of I–III and underwent elective surgery for uterine cancer. Exclusion criteria were renal or hepatic impairment, immunosuppressive therapy, immune system disorders, or cancer metastasis. Informed consent was obtained from all patients before participating in this study.

### Investigation

In total, 100 patients were enrolled and randomly assigned into one of the study groups (50 patients each) using a computer-generated random numbers table. In the dexmedetomidine group (DEX group), dexmedetomidine was infused at 0.4 μg/kg/h from anesthetic induction to the end of surgery and continued at 0.15 μg/kg/h for the first 24 h postoperatively. The dose of dexmedetomidine was determined based on that of previous studies showing no hemodynamic instability or deep sedation (4, 11). In the Control group, saline was infused at the same rates. One researcher prepared dexmedetomidine (Precedex; Hospira Inc, Lake Forest, IL, USA) or saline in identical 50-mL syringes labelled as “study drug” for double-blind purposes. Patients, surgeons, and anesthesiologists were blinded to the group assignment, which was revealed after participants were discharged from the hospital.

### Anesthetic Management

After monitors including electrocardiography, pulse oximetry and blood pressure monitor were applied, anesthesia was induced with propofol 1–2 m/kg and remifentanil 1–2 μg/kg. Rocuronium 0.6 mg/kg was administered to facilitate endotracheal intubation. Anesthesia was maintained with 4%–7% desflurane and remifentanil 0.05–0.1 μg/kg/min to maintain the mean arterial pressure within 20% of the preoperative value and the bispectral index between 40 and 60. Body temperature was maintained at 36.5 ± 0.5°C throughout surgery. At 15 min before the end of surgery, all patients received fentanyl 50 µg and ramosetron 0.3 mg for prevention of postoperative pain and nausea/vomiting. At the end of surgery, patients received neostigmine 1 mg and glycopyrrolate 0.2 mg for reversal of residual neuromuscular blockade. For postoperative analgesia, all patients received intravenous patient-controlled analgesia (IV-PCA) consisting of fentanyl 15 μg/kg and ramosetron 0.3 mg (total volume of 100 mL, basal rate of 2 mL/h, bolus of 0.5 mL, and lockout time of 15 min). Intravenous ketorolac 30 mg was administered three times per day on the day of surgery. Additional analgesics were available for patients having an 11-point numeric pain rating scale score ≥4 or requesting supplemental analgesics: intravenous fentanyl 50 μg or pethidine 25 mg in the post-anesthesia care unit and pethidine 25 mg or tramadol 50 mg in the postoperative ward. Drugs possessing anti-inflammatory effects (e.g., lidocaine, dexamethasone) were not administered during the first 48 h postoperatively. An investigator unaware of the group assignment evaluated possible dexmedetomidine-related adverse effects (e.g., deep sedation, hypotension, bradycardia).

### Outcome Measures

The primary outcome measure was NK cell activity, which was measured preoperatively and on postoperative days (PODs) 1, 3, and 5. NK cell activity was analysed using the NK Vue kit (ATGen, Sungnam, Korea). One mL of whole blood was drawn into a NK Vue tube containing Promoca (a cytokine that stimulates NK cell activity) and RPMI 1640 media and then incubated at 37°C for 24 h. This selected stimulatory cytokine and incubation period allows NK cells to secrete interferon-γ (IFN-γ) preferentially over other immune cells, and the supernatant IFN-γ level measured by NK Vue ELISA may be an indicator of NK cell activity. We calculated the mean IFN-γ value from duplicate readings.

Other outcome measures included inflammatory responses assessed by interleukin-6 (IL-6), IFN-γ, and neutrophil-lymphocyte-ratio (NLR), which were measured preoperatively and on POD 1, 3, and 5. Pain severity and opioid requirement were assessed 1, 24, and 48 h postoperatively. Pain severity was evaluated using an 11-point numerical scale (0 = no pain, 10 = worst symptom). The opioid requirement was assessed by IV-PCA fentanyl dose and additional opioid consumption (morphine equivalent dose). Rates of cancer recurrence or metastasis and death were assessed 2 years after surgery.

### Statistical Analysis

The sample size was calculated based on a previous study showing a reduction of NK cell activity on POD 1 (compared with baseline) of 83.1 ± 25.2% (12). Forty-eight patients in each group would be required to detect a 20% relative decrease in NK cell activity reduction with 90% probability (ß=0.1) at a significance level (α) of 0.05. Assuming a 5% dropout rate, the final sample size was 50 patients per group.

Continuous variables were analysed using the independent *t*-test or Mann-Whitney U test after testing for normality of distribution using the Kolmogorov-Smirnov test and expressed as mean ± SD or median (interquartile range). Categorical variables were analysed using χ^2^ or Fisher exact tests and expressed as absolute number (percentage). Variables measured repeatedly, such as NK cell activity, INF-γ, IL-6, and NLR, were analysed using a linear mixed model, with patient indicator as the random effect and group, time, and group-by-time as the fixed effects, after log-transformed for normality of distribution. *Post-hoc* analyses with Bonferroni correction were performed when variables measured repeatedly showed significant differences between groups. A *P* value <0.05 was considered statistically significant. Statistical analyses were performed with the Statistical Package for Social Sciences (SPSS 25.0, IBM Corp., Armonk, NY, USA).

## Results

**Figure 1:**
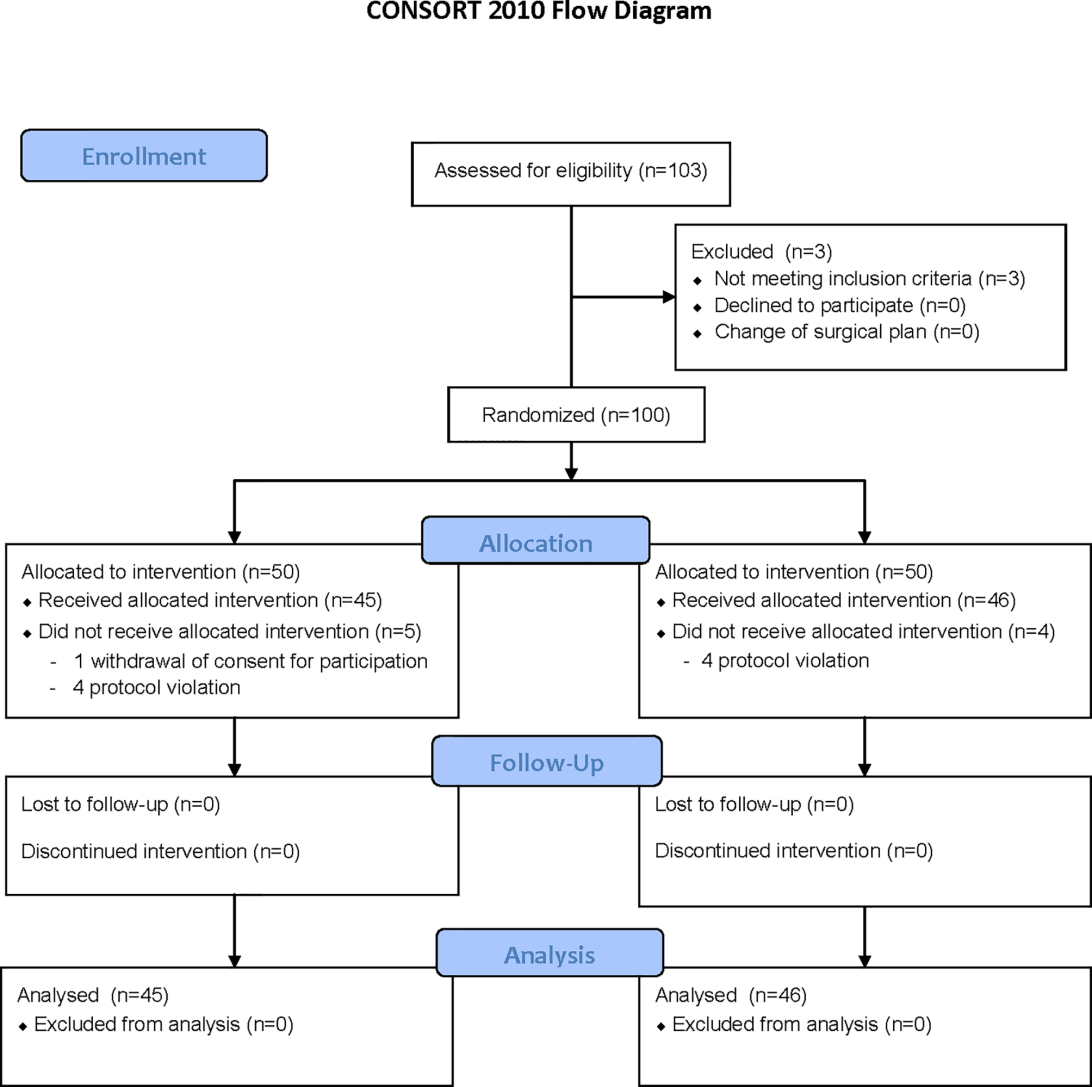
Consort diagram.

Of 100 patients enrolled, 9 patients were eliminated. One patient in the Control group withdrew consent for participation and 4 patients did not meet the study protocol (they were anesthetized with propofol or sevoflurane instead of desflurane). The remaining 91 patients completed the study without any complications (**Figure 1** and **Table 1**).

**Table 1:** Patient characteristics and operation details.

Variables	Control group (n = 45)	DEX group (n = 46)	*P* value
age (years)	52.2 (8.2)	51.4 (9.6)	0.641
body mass index (kg/m)	25.2 (3.9)	23.8 (3.4)	0.067
diabetes mellitus	8 (17.8%)	7 (15.2%)	0.742
ASA class I/II/III	21/13/11	24/17/4	0.135
cancer type			
cervix	9 (20.0%)	16 (34.8%)	0.201
endometrium	33 (73.3%)	29 (63.0%)	
myosarcoma	3 (6.7%)	1 (2.2%)	
operation			
total hysterectomy	14 (31.1%)	8 (17.4%)	
total hysterectomy with salpingo-oophorectomy	19 (42.2%)	20 (43.5%)	0.240
radical hysterectomy	12 (26.7%)	18 (39.1%)	
lymph node sampling	7 (15.6%)	5 (10.9%)	0.509
Cancer (FIGO) stage I/II/III/IV;	35/1/6/3	41/3/2/0	0.091
Preoperative neoadjuvant therapy	0	0	
Postoperative chemotherapy	16 (35.6%)	9 (19.6%)	0.088
Postoperative radiotherapy	15 (33.3%)	12 (26.1%)	0.449
Postoperative hormone therapy	0	0	
duration of operation (min)	194.3 (82.0)	175.2 (60.8)	0.211
duration of anesthesia (min)	230.1 (85.0)	209.0 (62.0)	0.180
propofol (mg/kg)	1.4 (0.3)	1.4 (0.2)	0.477
remifentanil (μg/kg/min)	0.06 (0.02)	0.05 (0.02)	0.004
bleeding (ml)	50 (20–100)	50 (30–100)	0.913
patients receiving erythrocyte transfusion	3 (6.7%)	2 (4.3%)	0.628

Values are mean (standard deviation), number (percent), or median (interquartile range). ASA class, American Society of Anesthesiologists physical status classification; FIGO staging, International Federation of Gynecology and Obstetrics staging.

### Natural Killer Cell Activity

**Figure 2:**
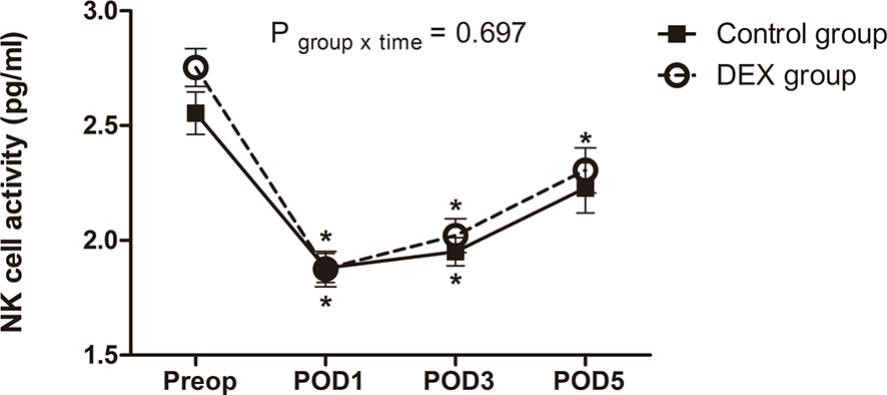
The change of natural killer cell activity. The perioperative change in natural killer cell activity was similar between groups (*P* = 0.697). Data was analyzed using a linear mixed model after log-transformation for normality of distribution. NK cell, natural killer cell; Preop, preoperatively; POD, postoperative day; DEX group, dexmedetomidine group; ^*^ *P* < 0.05 compared to preoperatively.

NK cell activity before surgery was comparable between groups (*P*=0.113) and it decreased significantly below baseline after surgery in both groups. Linear mixed model analysis showed that the perioperative change of NK cell activity over time was not different between groups (*P*=0.697) (**Figure 2**).

### Inflammatory Responses Measured by IFN-γ, IL-6, and NLR

IFN-γ level before surgery was comparable between groups (*P*=0.777). Compared to the baseline, IFN-γ level increased after surgery and was higher on PODs 3 and 5 in the DEX group, whereas it was maintained in the Control group. The change of IFN-γ over time was statistically significant between groups (*P*=0.010). IFN-γ level on POD 3 was higher in the DEX group compared to the Control group.

IL-6 level before surgery was lower in the DEX group than in the Control group (*P*=0.002). In both groups, IL-6 increased after surgery, peaking on POD 1. Compared to the baseline, IL-6 level was higher on PODs 1, 3, and 5 in the DEX group and on POD 1 in the Control group. The change of IL-6 over time was not significant between groups (*P*=0.117).

**Figure 3:**
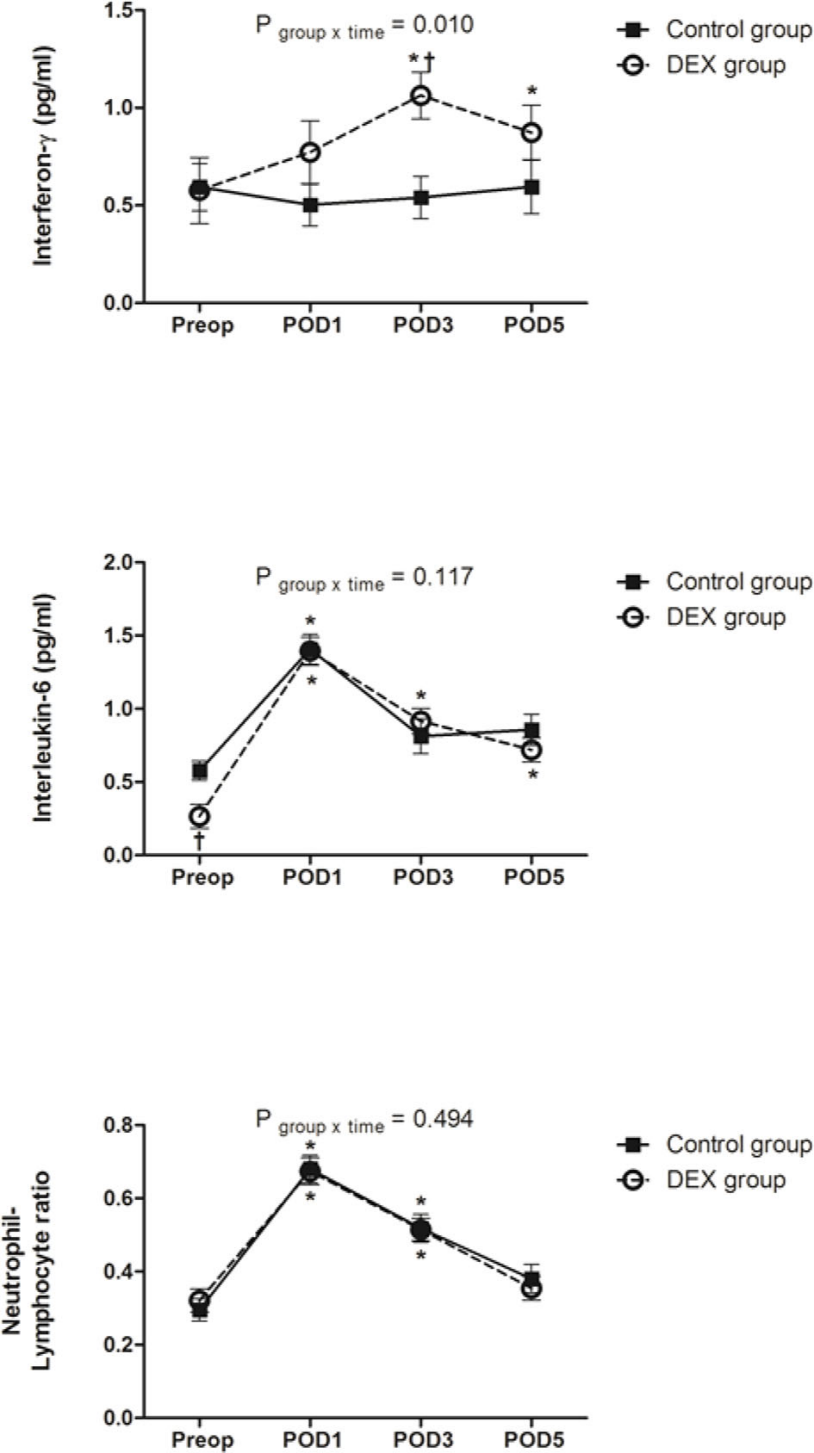
The change of interferon-γ, interleukin-6, and neutrophil/lymphocyte ratio. Change in interferon-γ over time was significantly different between groups (*P* = 0.010), whereas changes in interleukin-6 and neutrophil/lymphocyte ratio were similar between two groups. Data was analyzed using a linear mixed model after log-transformation for normality of distribution. Preop, preoperatively; POD, postoperative day; DEX group, dexmedetomidine group; ^*^ *P* < 0.05 compared to preoperatively; ^†^ *P* < 0.05 compared to the Control group.

NLR before surgery was similar between groups. It increased after surgery and was higher than baseline on PODs 1 and 3 in both groups. The change of NLR was not different between groups (*P*=0.494) (**Figure 3**).

### Pain Score and Opioid Consumption

Pain score during activity (sitting up) 1 h postoperatively was lower in the DEX group than in the Control group (3 [3–3] vs. 3 [3–5], *P*=0.016). At other times, pain scores were not different between groups. Fentanyl IV-PCA dosage during the first 48 h postoperatively was comparable between groups. Additional opioid consumption 1–24 h postoperatively was lower in the DEX group than in the Control group (3.3 [3.3–5.5] mg vs. 8.3 [5.0–10.8] mg, *P*=0.031). At other times, additional opioid consumption (converted to morphine equivalent) was similar between groups (**Table 2**).

**Table 2:** Pain scores and additional analgesic requirements.

Variable/ Time points	Control group (n = 45)	DEX group (n = 46)	*P* value
**Pain score (resting/ activity)**			
at 1 h after surgery	3 (3–3)/ 3 (3–5)	3 (2–3)/ 3 (3–3)	0.339/ 0.016
at 24 h after surgery	2 (0–3)/ 4 (2–5)	2 (0–4)/ 3 (2–5)	0.888/ 0.629
at 48 h after surgery	2 (1–3)/ 3 (2–5)	2 (0–3)/ 3 (2–5)	0.493/ 0.553
**Fentanyl administered via intravenous patient-controlled analgesia (μg)**			
0–24 h after surgery	462.8 (157.8)	402.2 (148.8)	0.064
24–48 h after surgery	296.6 (188.7)	303.9 (202.2)	0.860
**Additional opioid analgesics requirement (morphine equivalent dose, mg)**			
0–1 h after surgery	4.0 (3.2–5.0) (13)^*^	4.0 (3.0–4.0) (7)^*^	0.304 (0.102)
1–24 h after surgery	8.3 (5.0–10.8) (13)^*^	3.3 (3.3–5.4) (18)^*^	0.031 (0.303)
24–48 h after surgery	5.0 (3.3–10.0) (12)^*^	5.0 (5.0–8.3) (20)^*^	0.744 (0.093)

Values are median (interquartile range), mean (standard deviation), or number. Pain score, a numerical pain intensity scale (0 = no pain, 10 = the worst pain); ^*^, number of patients receiving additional opioids.

### Prognosis

Four patients (8.7%) in DEX group and 7 patients (16.3%) in the Control group had cancer recurrence and/or metastasis during the 2-year follow-up period (*P*=0.277). Death occurred in 1 patient (2.2%) in the DEX group (due to cancer recurrence with lung metastasis) and 3 patients (6.7%) in the Control group (due to cancer recurrence in 1 patient and cancer recurrence with lung metastasis in 2 patients) (**Table 3**).

**Table 3:** Prognosis.

Time points	Control group (n = 45)	Dex group (n = 46)	*P* value
**Recurrence and/or Metastasis**			
6 months after surgery	1 (2.2%)	0	0.309
1 year after surgery	6 (13.3%)	3 (6.5%)	0.276
2 years after surgery	7 (16.3%)	4 (8.7%)	0.277
**Death**			
6 months after surgery	0	0	
1 year after surgery	0	0	
2 years after surgery	3 (6.7%)	1 (2.2%)	0.318

Values are number (percent).

## Discussion

### Main Finding

Dexmedetomidine administration in patients undergoing uterine cancer surgery did not demonstrate a favorable impact on immunity in terms of perioperative changes of NK cell activity, IL-6, and NLR. However, dexmedetomidine was associated with higher IFN-γ postoperatively and reduced both pain severity and opioid requirement early postoperatively. Although statistically insignificant, rates of cancer recurrence/metastasis (8.7 vs. 16.3%) and death (2.2 vs. 6.7%) within 2 years after surgery were much lower in the dexmedetomidine group.

### Immunomodulation Effects of Dexmedetomidine

Major oncologic surgeries with extensive resection impair immunity, by causing sympathetic hyperactivation and excessive inflammation (13). It is important to carefully select anesthetics and analgesics not to aggravate perioperative immunosuppression. Theoretically, dexmedetomidine has beneficial effects on immunomodulation. Dexmedetomidine reduces surgical stress and inflammatory responses and attenuates the releases of catecholamines, cortisol, and pro-inflammatory cytokines (3). It also has analgesic and opioid-sparing effects and reduced postoperative pain and opioid consumption in major surgery, including cancer surgery (6, 14). A recent meta-analysis concluded that dexmedetomidine preserves immune function of surgical patients, decreases postoperative complications, and improves clinical outcomes (15). In addition, recent evidence indicates that α2A adrenoreceptors are involved in the progression of several malignancies, including breast, hepatocellular, and cervical cancers (16–18). α2A adrenoreceptor expression was significantly downregulated, which was associated with poor prognosis in cervical cancer patients (18). α2A adrenoreceptors suppressed cell proliferation, migration, and invasion and promote cell senescence and apoptosis, suggesting that this receptor might be a tumor-suppressor protein in cervical cancer (18). Thus, as a highly selective and potent α2A adrenoreceptor agonist, dexmedetomidine may be expected to exert beneficial immune effects in cervical cancer patients.

### Natural Killer Cell Effects

Perioperative immune dysfunction includes profound suppression of cell-mediated immunity, expressed as a decrease in the number and activity of immunocompetent cells such as NK and T cells (8). NK cells are a critical component of innate immunity and the main defence against cancer cell spread (19). A decrease in NK cell activity was associated with increased risk of mortality in patients undergoing cancer surgery (1). Adrenaline receptors are present in immune cells, and adrenergic mechanisms play an important role in regulating innate immunity (20). Cell-intrinsic adrenergic signalling is required for NK cells to exhibit optimal adaptive features during their responses against pathogens (21). Dexmedetomidine may affect NK cell activity by reducing the stress responses through sympatholytic action (3) and affecting α2 adrenoreceptors expressed in NK cells themselves. Few studies have examined the effects of dexmedetomidine on NK cells in cancer surgery patients. Dexmedetomidine attenuated the decrease in number of NK cells in patients undergoing radical mastectomy or brain neoplasm surgery (22, 23). Whereas previous studies measured NK cell number, we measured NK cell activity as an activity rather than a number should be a more reliable indicator of NK cell function. In the present study, dexmedetomidine did not attenuate postoperative suppression of NK cell activity in patients undergoing uterine cancer surgery. Further studies are necessary to clarify the effects of dexmedetomidine on NK cell function by measuring both number and activity.

### Inflammation Effects

Exaggerated inflammatory responses with excessive production of proinflammatory cytokines induced by surgical trauma also contribute to immune dysfunction (8). Dexmedetomidine modulates cytokine production by macrophages and monocytes and activates cholinergic anti-inflammatory pathways by stimulating α2 adrenoreceptors. Dexmedetomidine has been well demonstrated to exert anti-inflammatory properties and reduce the release of proinflammatory cytokines, such as IL-6, tumor necrosis factor-α, and C-reactive protein, in major surgery (4, 14). Contrary to the findings that dexmedetomidine attenuated the early postoperative increase in IL-6 after radical gastric or colon cancer surgery (5, 14), there was no difference in the changes of IL-6 between our study groups. These discrepant results may be attributed to the time points of IL-6 measurement, which were measured later in our study (PODs 1, 3, and 5) than in previous studies (at the end of surgery and 24 h postoperatively). In patients undergoing gastric cancer surgery, IL-6 at 48 h after surgery did not differ between control and dexmedetomidine groups (14).

Interestingly, dexmedetomidine was associated with a higher IFN-γ level postoperatively. IFN-γ is produced by activated T cells and NK cells in response to immune stimuli and enhances cellular immune immunity (24). It exerts both anti- and pro-tumorigenic effects. IFN-γ signalling inhibits tumor growth by inducing tumor cell apoptosis and necrosis, producing tumor ischemia, and activating antigen-presenting and effector cells, while inhibiting suppressive immune cells (25, 26). On the other hand, IFN-γ exerts feedback inhibitory effects by suppressing over-activation of the immune system, which is related to immune escape from the tumor microenvironment and contributes to tumor growth (25). IFN-γ-producing capability was impaired in patients with invasive cervical cancer (27). IFN-γ genetic polymorphisms increased the risk of cervical cancer (28), and low levels of intra-tumoral IFN-γ mRNA was associated with poor prognosis (29). IFN-γ inhibits the proliferation of endometrial carcinoma cells (30). In the present study, dexmedetomidine significantly increased IFN-γ levels, which was not accompanied by a favorable impact on NK cell activity. Our finding is in line with a previous study reporting no clear association between IFN-γ gene expression and NK cell infiltration in invasive cervical carcinoma (29). Although tumor-infiltrating NK cells and T cells are the main sources of IFN-γ, several factors also regulate IFN-γ expression, including lactic acidosis, epigenetic modifications, and microRNA-155 (25). Further investigations are required to determine whether dexmedetomidine-induced increases in IFN-γ have beneficial effects on clinical outcomes in cancer surgery.

### Pain Effects

Pain suppresses NK cell activity directly and indirectly by activating the sympathetic nervous system and increasing the secretion of catecholamine (31, 32). Although opioid is essential for analgesia after cancer surgery, it suppresses immunity by acting on the µ-opioid receptor expressed in immune cells and indirectly *via* the hypothalamic-pituitary-adrenal axis (33, 34). Based on these theoretical basis, dexmedetomidine may help preserve immune function by reducing pain and opioid requirement and suppressing sympathetic activation (3). In the present study, dexmedetomidine reduced both postoperative pain and opioid consumption in the early postoperative period. Pain severity with activity during the first 1 h and additional opioid consumption during the first 1–24 h after surgery were lower in the DEX group.

### Limitations

This study has several limitations. First, different types of uterine cancer were included, which might have influenced the immune and inflammatory responses and prognosis, although the cancer types were comparable between the groups. Second, intraoperative remifentanil concentration was higher in the Control group, and thus its potential effects on immunity cannot be excluded. However, remifentanil in clinically relevant doses did not impair NK cell function (35). Third, rates of cancer recurrence/metastasis (16.3% vs. 8.7%) and death within 2 years after surgery (6.7% vs. 2.2%) were 2 times higher in the Control group than in the DEX group, but there was no statistical difference. As the sample size might have been insufficient to detect differences in these secondary outcomes, the association between dexmedetomidine and recurrence/metastasis cannot be concluded from our results. To clarify the effect of dexmedetomidine on cancer prognosis, further study with this as a primary outcome is needed.

## Conclusion

Perioperative administration of dexmedetomidine did not preserve NK cell activity in patients undergoing uterine cancer surgery. It did not affect the inflammatory responses, cancer recurrence/metastasis rate, and mortality. However, dexmedetomidine had favourable effects of increasing IFN-γ and reducing early postoperative pain and opioid consumption.

## Data Availability Statement

The raw data supporting the conclusions of this article will be made available by the authors, without undue reservation.

## Ethics Statement

The studies involving human participants were reviewed and approved by the Institutional Review Board and Hospital Research Ethics Committee of Severance Hospital, Yonsei University Health System, Seoul, Korea (#4-2015-0453) and registered at ClinicalTrials.gov (NCT02896413). The patients/participants provided their written informed consent to participate in this study.

## Author Contributions

JC: Conceptualization, Funding acquisition, Investigation, Data curation, Formal analysis, Writing- original draft. KS: Investigation, Data curation, Formal analysis. M-YK: Investigation, Data curation, Formal analysis. SK and YY: Conceptualization, Supervision, Writing - review & editing. JC, MK, SK, and YY: Agreement to be accountable for all aspects of the work. All authors contributed to the article and approved the submitted version.

## Funding

This study was supported by a faculty research grant of Yonsei University College of Medicine (6-2016-0107).

## Conflict of Interest

The authors declare that the research was conducted in the absence of any commercial or financial relationships that could be construed as a potential conflict of interest.

## Publisher’s Note

All claims expressed in this article are solely those of the authors and do not necessarily represent those of their affiliated organizations, or those of the publisher, the editors and the reviewers. Any product that may be evaluated in this article, or claim that may be made by its manufacturer, is not guaranteed or endorsed by the publisher.
