# Supplementary figures and images for: Effects of Perioperative Dexmedetomidine on Immunomodulation in Uterine Cancer Surgery: A Randomized, Controlled Trial
Source: Front Oncol. 2021 Nov 16;11:749003. doi: 10.3389/fonc.2021.749003 (PMC8635094; doi:10.3389/fonc.2021.749003)

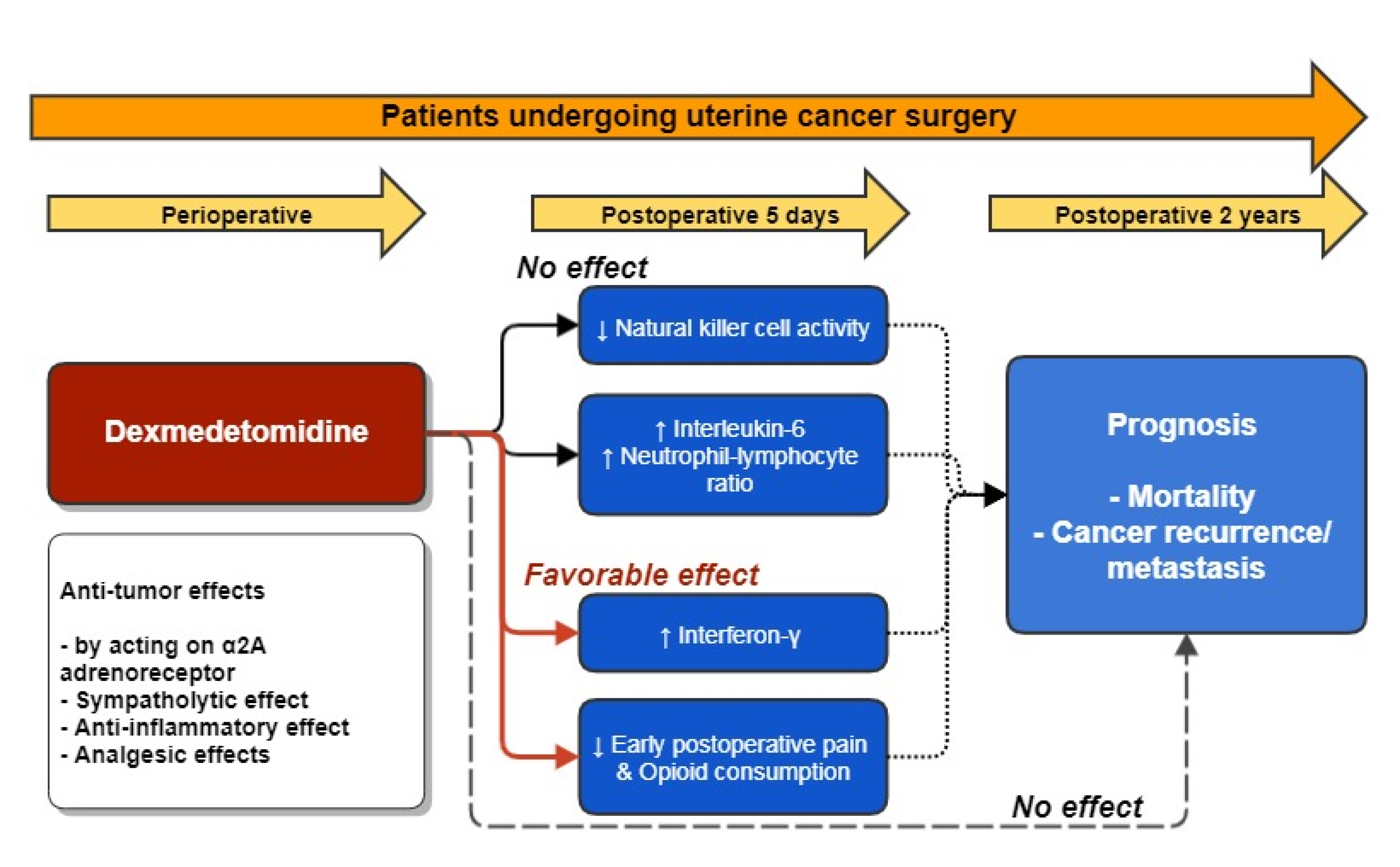

Supplement: Supplementary file 1 [file Image_1.tif]
